# Solid lipid-based nanoparticulate system for sustained release and enhanced *in-vitro* cytotoxic effect of 5-fluorouracil on skin Melanoma and squamous cell carcinoma

**DOI:** 10.1371/journal.pone.0281004

**Published:** 2023-02-28

**Authors:** Ahsan Ali, Asadullah Madni, Hassan Shah, Talha Jamshaid, Nasrullah Jan, Safiullah Khan, Muhammad Muzamil Khan, Muhammad Ahmad Mahmood

**Affiliations:** 1 Department of Pharmaceutics, Faculty of Pharmacy, The Islamia University of Bahawalpur, Bahawalpur, Pakistan; 2 Akson College of Pharmacy, Mirpur University of Science and Technology (MUST), Mirpur, AJ&K, Pakistan; 3 Cadson College of Pharmacy, Kharian, Pakistan; Central University of Rajasthan, INDIA

## Abstract

The present study aimed to prepare solid lipid-based nanoparticles (SLNs) using Precirol^®^ ATO 5 as solid lipid and Poloxamer 188 and Tween 80 as surfactant and co-surfactant respectively, and SLNs-derived gel for sustained delivery, enhanced *in-vitro* cytotoxicity, enhanced cellular uptake of 5-FU and enhanced permeation of 5-FU across the skin. The 5-FU-loaded SLNs were prepared by the hot melt encapsulation method and converted into SLN-derived gel using a gelling agent (Carbopol 940). The 5-FU-loaded SLNs had a particle size in the range of 76.82±1.48 to 327±4.46 nm, zeta potential between -11.3±2.11 and -28.4±2.40 mV, and entrapment efficiency (%) in range of 63.46±1.13 and 76.08±2.42. The FTIR analysis depicted that there was no chemical interaction between 5-FU and formulation components. Differential scanning calorimetric analysis showed thermal stability of 5-FU in the nanoparticles and powdered X-ray diffraction analysis revealed successful incorporation of 5-FU in nanoparticles. The *in-vitro* release study of 5-FU-loaded SLNs showed biphasic release behavior with initial burst release followed by sustained release over 48 hr. The 5-FU-loaded SLNs showed a greater cytotoxic effect on skin melanoma (B16F10 cells) and squamous cell carcinoma (A-431 cells) as compared to free 5-FU drug solution after 48 hr. Flow cytometry and fluorescence microscopy displayed enhanced quantitative and qualitative cellular uptake of SLNs. The SLNs formulation showed acceptable safety and biocompatible profile after an acute toxicity study in Wistar rats. Moreover, *ex-vivo* permeation studies depicted 2.13±0.076 folds enhanced flux of 5-FU-loaded SLN derived gel compared to 5-FU plain gel, and skin retention studies revealed target efficiency (%) 2.54±0.03 of 5-FU-loaded SLN derived gel compared to 5-FU plain gel.

## 1. Introduction

Skin cancer is one of the most common cancers throughout the globe [1, 2]. On daily basis, about 9,500 people are diagnosed with skin cancer in the United States [3]. The main skin malignancies are melanoma, and squamous cell carcinoma (SCC). Research estimates that SCC affects more than 3 million Americans every year [4], and more than 1 million Americans are living with melanoma [5]. Skin cancer is usually caused due to excessive and prolonged exposure to direct sunlight because it leads to the destruction of keratinocytes and cellular proliferation [6]. Common treatment protocols for skin cancers include surgical excision or radiation therapy [7, 8]. In addition, chemotherapy might be a better alternative in some situations where surgery is contraindicated or not feasible.

5-FU is one of the most common chemotherapeutic agents which is being used against many cancer types including pancreatic cancer, breast cancer, colorectal cancer, and many topical diseases including actinic keratosis and skin cancers [9]. 5-FU induces its cytotoxic effect on cancer cells by inhibiting cellular thymidylate synthase enzyme, the inhibition of which leads to failure of the DNA replication process and inhibits RNA synthesis by integrating its metabolites into RNA after intracellular activation [10, 11]. However, the delivery of 5-FU to the cancer site is an issue due to low skin permeability because of its hydrophilic nature and unfavorable hydrophilic/lipophilic balance [12, 13]. Using conventional drug delivery systems result in less-than-optimal delivery of 5-FU to treat skin cancer, and this has made it necessary to use other measures to obtain therapeutic outcomes [14, 15]. Several lipid nanoparticulate delivery systems have been exploited to enhance 5-FU skin permeability [16, 17]. Among these nanoparticulate drug delivery systems, SLNs have been fabricated as an alternative to other novel drug delivery systems because of having advantages like the feasibility of incorporating lipophilic and hydrophilic drugs, improved physical stability, low cost, and ease of scale-up and manufacturing [18].

SLNs prepared with biocompatible and biodegradable lipids usually have a size range of about 50–1000 nm resulting in good storage stability of drugs [19]. So in recent years, much work has been done in the development of SLNs for the delivery of anti-cancer drugs, peptides, genetic material, cosmetics, etc. [20–22]. SLNs not only improve the entrapment and loading of chemotherapeutic agents like 5-FU but also reduces the toxicity of drugs to normal cells [18]. Moreover, SLNs are an ideal candidate for topical delivery due to their potential for epidermal targeting, follicular delivery, sustained drug delivery, skin hydration, and photostability [23–25]. Furthermore, there is the possibility to produce SLNs on a large scale at a low cost with a simple method that uses high-pressure homogenization [26].

In the present study, 5-FU-loaded SLNs were fabricated using Precirol^®^ ATO 5 as solid lipid and Poloxamer 188 and Tween 80 as surfactant and co-surfactant respectively, to sustain the delivery, enhance permeability of 5-FU, *in-vitro* cytotoxicity against murine melanoma B16F10 and human A431 squamous cell carcinoma cell lines, and cellular uptake of 5-FU-loaded SLNs.

## 2. Materials and methods

### 2.1. Materials

Precirol^®^ ATO 5 (Glyceryl palmitostearate 100%) was provided as a kind gift sample from Gattefosse (NJ, USA). Poloxamer 188 was purchased from Merck (Germany). Tween 80, ethanol (95%), and 5-FU (≥99%) were purchased from Sigma-Aldrich Co. (St Louis, MO, USA). Dialysis bags (MWCO: 10K Da) were purchased from Spectrum Labs (Rancho Dominguez, Canada). Ethanol was purchased from Merck KGaA, (Germany). The skin melanoma cell lines (B16F10) and squamous cell carcinoma cell lines (A-431) were obtained from ATCC, USA.

Dulbecco’s Modified Eagle’s Medium (DMEM), Penicillin-streptomycin, phosphate buffer saline (PBS), and fetal bovine serum (FBS) werepurchased from Thermo Fisher Scientific (Waltham, MA, USA). Cell TiterBlue^®^ was purchased from Promega^®^ (WI, USA). All other chemicals and reagents used for the study were of analytical grade. Deionized water was used throughout the experiment.

### 2.2. Preparation of 5-FU-loaded SLNs

SLNs were prepared by the hot melt encapsulation (HME) method as reported in the literature with modifications [27, 28]. 5-FU-loaded SLNs were fabricated with varying concentrations of solid lipid and surfactant, whereas the concentration of drug and co-surfactant was kept constant (Table 1). Briefly, Precirol^®^ ATO 5 was melted to 5°C above its melting point and 5-FU was added to it. Stirring was continued for 5 minutes, and an aliquot of ethanol was added to facilitate the homogenous mixing of 5-FU (5mg) in the lipid phase. The aqueous phase (5 mL) was prepared by mixing Poloxamer 188 (surfactant 1%-3%) and Tween 80 (co-surfactant 0.5%) in water and heated to the same temperature used for the preparation of the lipid phase. Then, the lipid phase was added dropwise to the aqueous phase and stirring was continued. The mixture was then homogenized for 3 minutes at 12000 rpm using a homogenizer (Polytron PT 1200E, Germany). After 5 minutes, the heater of the hot plate stirrer was switched off and the hot melt emulsion so formed was allowed to cool down to room temperature under continuous stirring followed by sonication for 5 minutes. SLNs dispersion was centrifuged at 14000 rpm for 20 minutes at room temperature and washed with deionized water. Finally, SLNs were lyophilized at -45°C and reduced pressure for 24 hr.

**Table 1:** Physicochemical characteristics of 5-FU-loaded SLNs.

Code	Precirol^®^ ATO 5 (mg)	Poloxamer 188: Tween 80 (%w/v)	Size (nm)	PDI	Zeta Potential (mV)	%EE
SLN1	100	3%: 0.5%	327± 4.5	0.442±0.009	-9.66± 1.2	70.60%± 1.2
SLN2	100	2%: 0.5%	220.3± 3.3	0.430±0.007	-11.3± 2.1	67.72%± 2.1
SLN3	100	1.5%: 0.5%	125.3± 4.8	0.354±0.013	-20.4± 1.5	63.46%± 1.1
SLN4	100	1%: 0.5%	100.3± 2.9	0.257±0.006	-28.4± 2.6	76.08%± 2.4
SLN5	75	1%: 0.5%	84.09±1.01	0.345±0.004	-21.3± 1.2	73.40%± 3.2
SLN6	50	1%: 0.5%	76.82± 1.5	0.356±0.008	-19.4± 2.4	71.16%± 2.9

Data was presented as mean ±SD (n = 3)

### 2.3. Preparation of 5-FU-loaded SLNs gel

The 5-FU-loaded SLNs gel was prepared by dispersing 5-FU-loaded SLNs dispersion equivalent to 2 mg of drug in 1% w/v Carbopol-940 solution, followed by stirring at 1000 rpm for 2 hr. Triethanolamine (0.9%) was added for gel consistency and to adjust pH. The 5-FU plain gel was also prepared by the same method for comparison with the 5-FU-loaded SLNs gel [29].

### 2.4. Characterization of 5-FU-loaded SLNs

#### 2.4.1. Particle size, zeta potential, and polydispersity index

The particle size, zeta potential, and polydispersity index (PDI) of fabricated SLNs were determined by dynamic light scattering (DLS) technique using a Zeta Sizer-ZS90 (Laser power 4mW, 633nm, Malvern Instrument, Worcestershire, UK) at a fixed angle of 90°C, and at temperature 25°C. Analysis of all the samples was done in triplicate and data was presented in mean ± S.D (n = 3).

#### 2.4.2. Entrapment Efficiency (%EE)

%EE of 5-FU-loaded SLNs was determined by an indirect method [30]. The 5-FU-loaded SLNs were prepared and centrifuged at 12,000 rpm. The supernatant was used to measure the amount of un-entrapped drug in the nanoparticles. The amount of un-entrapped drug was measured using a UV/Visible spectrophotometer at 266 nm [31]. The %EE of 5-FU in the developed SLNs was determined by the following formula:

$$\%\text{EE}=\left(\text{Weight}\;\text{of}\;5\text{-FU}\;\text{added-Free}\;\text{amount}\;\text{of}\;5\text{-FU}\right)/\left(\text{Weight}\;\text{of}\;5\text{-FU}\;\text{added}\right)\times 100$$ (1)

#### 2.4.3. Morphology by transmission electron microscopy

The morphology of prepared 5-FU-loaded SLNs was analyzed by using the transmission electron microscope (Jeol, USA). The sample of 5-FU-loaded SLNs was applied directly to the copper grid of the microscope and suitable images were taken at different magnifications to investigate the morphology of the developed SLNs.

#### 2.4.4. Fourier transform infrared spectroscopic (FTIR) analysis

FTIR analysis was performed using ATR-FTIR (Bruker, ALPHA P Series, Germany) to determine any interaction between formulation components [32]. 5-FU, Precirol^®^ ATO 5, Poloxamer 188, Tween 80, physical mixture, and optimized 5-FU loaded SLNs formulation were scanned between 400 cm^-1^ and 4000 cm^-1^ at the scan resolution of 4 cm^-1^ and sample scan time of 64 scans to measure FTIR spectrum. The presence of characteristic peaks will confirm that each component is in physical contact and no chemical interaction is there. In case of any chemical interaction, shifting or loss of prominent peaks may happen. The appearance of a new peak in the FTIR spectra will indicate the formation of the new bond [33].

#### 2.4.5. Powdered x-ray diffraction (pXRD) analysis

Powdered x-ray diffraction analysis of 5-FU, Precirol^®^ ATO 5, Poloxamer 188, physical mixture of individual components, and Optimized formulation was performed by scanning from 10–60° (0.03°/0.7 seconds per step) using a Rigaku MiniFlex+, variable slit, diffractometer equipped with a Cu-target X-ray tube, operating at 30 kV/15 mA, and a simple nickel Kβ-filter and scintillation counter detection system. The generated X-ray data files were converted to text files (2°θ versus Xray counts per second) for further plotting.

#### 2.4.6. Thermal behavior by Differential Scanning Calorimetric (DSC) analysis

A differential scanning calorimeter (PerkinElmer Diamond DSC, CT) equipped with an intercooler 1P was used for thermal analysis of 5-FU, Precirol^®^ ATO 5, Poloxamer 188, physical mixture, and optimized SLNs formulation. The samples were sealed in a 20-μL aluminum pan and placed in DSC furnace. The thermographs were recorded by subjecting each sample to a thermal program at heating rate of 10°C/min in the temperature range between 20–300°C under nitrogen gas maintained at a flow rate of 20 mL/min [34].

#### 2.4.7. In-vitro drug release studies

*In-vitro* drug release studies from lyophilized 5-FU-loaded SLNs were performed in phosphate buffer saline (PBS) of pH 7.4. The accurately weighed lyophilized 5-FU-loaded SLNs (Weight equivalent to 5 mg of 5-FU) was dispersed with 3 mL of PBS pH 7.4 in the dialysis bag (MWCO: 10 k Da, soaked in distilled water for 12 hr prior study). The dialysis bags were clipped with clamps and immersed in a vessel of USP type II dissolution apparatus and filled with 200 mL of dissolution media (PBS of pH 7.4) solution at 37 ± 0.5°C and 50 rpm speed. 2mL samples were withdrawn at a pre-determined time interval and replaced with an equal volume of PBS (pH 7.4). Finally, the release of 5-FU in the dissolution medium was determined by using a UV–visible spectrophotometer at 266 nm [34, 35].

#### 2.4.8. Kinetic modeling

The dissolution data obtained was applied to the kinetic modeling using Zero order, First order, Higuchi model, and Korsmeyer–Peppas model. The values of the regression coefficient (R^2^) and release exponent (*n*) were analyzed for the mechanism of drug release from formulations using a DD solver.xla (Add-In of MS-Excel) [36].

### 2.5. Cell lines and cell culture

#### 2.5.1. Cell lines

Dulbecco’s Modified Eagle’s Medium (DMEM) containing 1500 mg/L sodium bicarbonate, 4mM L- glutamine, 4500 mg/L glucose, and 1mM sodium pyruvate was used to culture skin melanoma cells (B16F10 cell lines) and squamous cell carcinoma cells (A-431 cell lines). Fetal bovine serum, FBS (10%), and 10 IU/mL of streptomycin (antibiotics) were also added tomedium. The media was changed regularly, and cells were passaged twice per week.

#### 2.5.2 In-vitro cytotoxicity study

*In-vitro* cytotoxicity study was performed when cells reached 75% of confluency. Cells were collected by trypsinization (0.25% w/v trypsin-0.53Mm EDTA solution) and 7,000 cells per well were seeded in sterile, polystyrene 96-well plates (Costar^®^, USA). Cells were incubated in 5% CO_2_ atm at 37°C for 24 h. Four groups of wells were utilized in this study. One group had cells on which no treatment was applied and was considered as a control group. The second group of wells had cells that were treated with various aliquots of blank SLNs. The third group of wells had cells exposed to variable concentration 5-FU drug solution (400–12.5 μM), and the fourth group of wells had cells that were treated with 5-FU-loaded SLNs in the variable concentration of loaded 5-FU (400–12.5 μM). The treated cells were kept in an incubator for 24 hr and 48 hr at 37°C in an incubator. At the end of the incubation period, the viability of adherent cells was checked with Cell Titer-Blue^®^ assay (Promega, Madison, USA) and counted by the plate reader (BioTek) after 24 hr and 48 hr of treatment.

#### 2.5.3. Cell uptake studies

*2*.*5*.*3*.*1 Quantitative uptake by flow cytometry*. The quantitative cellular uptake study of nanoparticles was evaluated using flow cytometry (Beckton Dickinson FACS Calibur^™^, NJ, USA). B16F10 and A-431 cells (1 Million) were seeded in each well of a six-well plate and were incubated for 24 hr at 37°C and 5% CO_2_. After 24 hr incubation, cells were treated with SLNs containing Rh-PE (Rhodamine-PE 1 mol%) for 4 hours in serum complete media. Trypsin was used to detach the cells from the wells and after that cells were washed three times with PBS pH 7.4 and centrifuged at 1000 rpm for 5 min and resuspended in 200 μL PBS of pH 7.4. The fluorescence signal was obtained using a 488 nm laser and the emission was recorded using a 530/30 nm wavelength filter. A total of 10,000 gated live cell events were collected [37].

*2*.*5*.*3*.*2*. *Qualitative uptake by fluorescence microscopy*. For the qualitative uptake study of SLNs fluorescence microscopy technique was used. Briefly, B16F10 cells and A-431 (50,000) cells were seeded in 24 well plate using microscopic cover glass. After 24 hr incubation, cells were treated with SLNs loaded with Rh-PE for 4 hr. After 4 hr, the cells were washed with PBS (pH 7.4) and were fixed with PBS containing 4% of paraformaldehyde (PFA) for 30 min at room temperature. After 30 minutes, cells were again washed with PBS three times and stained with 10μg/mL Hoechst 33342 in PBS for 15 min. After washing again with PBS, cells were mounted on Fisherbrand Superfrost^®^ microscope slides with Fluoromount G^®^ mounting buffer (Southern Biotech, AL, USA) for analysis by fluorescence microscopy using KEYENCE (BZ-X710) fluorescence microscope.

### 2.6. Acute toxicity study

An acute toxicity study was performed to determine the safety and biocompatibility of developed blank SLN4 formulation according to Organization for Economic Co-operation and Development (OECD) guidelines [40]. The study was also approved by the Institutional Ethical Committee (Pharmacy Animal Ethics Committee, The Islamia University of Bahawalpur, Pakistan) with reference letter no. 33-2021/PAEC. In the present study, 12 Wistar rats were selected and divided into two groups (n = 6) weighing 180–200 gm labeled as control group and blank SLN4 test group. Separate neat and well-ventilated cages were used to place Wistar rats and supplied them with water and healthy food. The control group was given sterile water for injection whereas the blank SLN4 test group was given the developed blank SLN4 formulation (single dose of 2000 mg/kg) via the oral route. For 14 days the animals were observed for any change i.e., toxicity, mortality rate, and food and water consumption. On the 15th day, for biochemical analysis (i.e., hematological parameter, lipid profile, renal function test, and liver function test),the animals were anesthetized with ketamine and xylazine to avoid any distress/discomfort and the blood samples were taken and then rats were sacrificed using cervical dislocation method for histopathological examination of vital organs. [29].

### 2.7. Evaluation of 5-FU-loaded SLNs gel

#### 2.7.1. Physical appearance, pH, spreadability, and rheological analysis

Physical appearance includes smoothness, transparency, homogeneity, and color. The pH of 5-FU-loaded SLNs gel and 5-FU plain gel was measured by utilizing a pH meter (Mettler Toledo S-20 K, Switzerland) [38]. The spreadability of 5-FU plain gel and 5-FU-loaded SLNs gel was determined by using the glass slide method as reported earlier [39]. Briefly, a circle of 1 cm was marked on one glass slide and about 0.5 g of gel was placed on the marked area, and the glass slide was covered using another glass slide. A weight of 500 g was placed on the upper glass slide for 5 minutes. After 5 minutes the weight was removed and the increase in diameter was measured using a scale.

Brookfield Rheometer (Model DV-III, spindle number CP41, Brookfield engineering laboratories) was used to measure rheological parameters (viscosity, shear rate, and shear stress) of freshly prepared 5-FU plain gel and 5-FU-loaded SLNs gel at 25°C. A gel sample of 0.5 ± 0.03 g was placed in the sample holder and measurements were taken at speed ranging from 20–100 rpm [40].

#### 2.7.2. Ex-vivo permeation study

The *ex-vivo* permeation of 5-FU-loaded SLNs gel and 5-FU plain gel was determined using excised skin of Wistar rats. The *ex-vivo* permeability study was carried out by Franz diffusion cell (Perme Gear, Inc. No: 4G-01-00-15-12) with a volume capacity of 12 mL and surface area of the opening of 1.76 cm^2^ using hairless excised rat skin. The receptor compartment was filled with PBS of pH 5.5 maintaining experimental conditions of 300 rpm stirring speed and 37°C ± 2 temperature for 24 h. The samples were taken at predefined regular intervals and replaced with an equal volume of phosphate buffer to maintain sink conditions. The rate of drug permeation was determined through the graph plotted between the percent drug permeated across Wistar rat skin and various parameters of drug permeability such as flux and enhancement ratio were calculated [41, 42].

#### 2.7.3. Skin retention study

A skin retention study was performed by the reported method with some modifications [43]. Briefly, at the end of the permeation study, the excess part of the gel formulations was removed from the surface of the skin samples and the skin was rinsed with phosphate buffer solution and dried. The skin samples were frozen, cut into smaller pieces, and were then extracted with 20 mL of methanol by mechanical shaking in the water bath at 37 ± 1°C overnight. To further improve the extraction of 5-FU, skin samples were sonicated for 30 min. All the samples were harvested by ultracentrifugation (12,000 rpm) at 10°C for 20 min and quantification of 5-FU in rat skin was performed using a UV-visible spectrophotometer.

### 2.8. Statistical analysis

Origin Pro 8.5, GraphPad Prism 8, and Microsoft Excel 2019 were used for statistical analysis. Student t-test and ANOVA was applied to find the significant difference between groups and *p*-value <0.05 was considered statistically significant. Moreover, all the processes were performed in triplicate, and data was expressed as mean ± standard deviation [44].

## 3. Results

### 3.1. Physicochemical characterization of 5-FU-loaded SLNs

In the present study, the SLNs were prepared using the HME method which is superior to other methods due to the lack of toxic organic solvents and the ease of process parameters. Precirol^®^ ATO 5 has a diversity of fatty acids–and a looser structure helping it to entrap both hydrophilic and hydrophobic drugs, so it was selected to be used as lipid core for the preparation of 5-FU-loaded SLNs in this study [45, 46]. Poloxamer 188 and Tween 80 were used as surfactant and co-surfactant respectively to avoid aggregation of particles for stable formulation.

#### 3.1.1. Particle size, polydispersity index (PDI), and zeta potential

The nanoparticles having size around 200 nm provides higher drug concentration in tumor the microenvironment and reduce lymphatic drainage in tissue leading to increased therapeutic effect. This phenomenon is called the enhanced permeability and retention effect (EPR) [35]. The particle size may affect the drug release, entrapment efficiency, cytotoxicity, and pharmacokinetic behavior [5]. The mean particle size of lipid nanoparticles usually depends on several factors, such as the type and concentration of lipids and surfactants [13].

*3*.*1*.*1*.*1*. *Effect of concentration of poloxamer 188 on particle size*. The poloxamer 188 concentration has a prominent effect on particle size as also depicted by results in Table 1. In the first three formulations (SLN1, SLN2, and SLN3), the lipid ratio was kept constant while the variable concentration of poloxamer 188 was used (3%, 2%, 1.5% w/v). The particle size decreases with the decrease in the concentration of poloxamer 188 from 327±4.46 nm to 125.33±4.75 nm (Table 1) because when poloxamer 188 is used in higher concentrations it gets absorbed on the surface of nanoparticles leading to an increase in size. The same results of the effect of poloxamer on particle size have already been reported by Sanjula, B., et al., 2009 [47] and Khan, S., et al., 2021 [48]. So, the concentration of poloxamer 188 was optimized to 1% for SLN4, SLN5, and SLN6 as an optimal concentration of surfactant is needed for reduced particle size and stability of the nanoparticles [49].

*3*.*1*.*1*.*2*. *Effect of lipid concentration on particle size*. There is a direct relation between lipid concentration and the particle size of 5-FU-loaded SLNs. It was found that by decreasing the concentration of Precirol^®^ ATO 5 the size of 5-FU-loaded SLNs also decreases from SLN4 to SLN6 (100.3±2.86 nm to 76.82±1.48 nm) (Table 1), maintaining the concentration of surfactant and co-surfactant. Similar findings have already been reported by Abhijit A. Date et al., 2011 [50].

*3*.*1*.*1*.*3*. *Zeta potential and PDI*. The zeta potential is also important for the colloidal stability of the SLNs and for identifying the surface charge of nanoparticles [51]. The higher (positive or negative) zeta potential could cause strong repulsive forces among the nanoparticles indicating good stability [52]. All the 5-FU-loaded SLNs formulations were shown to have zeta potential between -11.3±2.11 mV and -28.4±2.40 mV, which is desirable in topical drug delivery applications [53].

PDI measures the size distribution of the nanoparticles in a sample [54]. The PDI value of all the batches of 5-FU-loaded SLNs formulations was found less than 0.5, suggesting that they were all monodispersed with uniform particle size distribution (Table 1).

#### 3.1.2 Entrapment efficiency

The entrapment efficiency of all the 5-FU-loaded SLNs formulations was presented in Table 1. In SLN1 to SLN3, it was observed that with the decrease in the concentration of poloxamer 188, EE also decreases from 70.60%±1.16 to 63.46%±1.13 because poloxamer 188 surfactant helps to solubilize and stabilize the drug molecule to get entrapped within the lipid matrix and at the surface of nanoparticles. This effect was found significant when t-tested was applied (*p<*0.05). The same effect of poloxamer 188 on EE has already been reported [55, 56].

From SLN4 to SLN6, it was observed that in presence of a sufficient concentration of surfactant and co-surfactant, an increase in the concentration of Precirol^®^ ATO 5 also resulted in increased EE, because the use of solid lipid causes enormous defects in crystal lattice resulting in higher imperfections, thus, enhancing the space to entrap drug molecules [57]. So, higher lipid content could prevent the escape of the drug to the outer environment by effectively enclosing the surfactant, which could be the possible reason behind the increase in the EE of SLNs. The effect was found significant when t-tested (*p<*0.05). The same results have been reported by Nazemiyeh et al., 2016 [58], and Khames et al., 2019 [59].

#### 3.1.3. Morphology by TEM analysis

**Fig 1:**
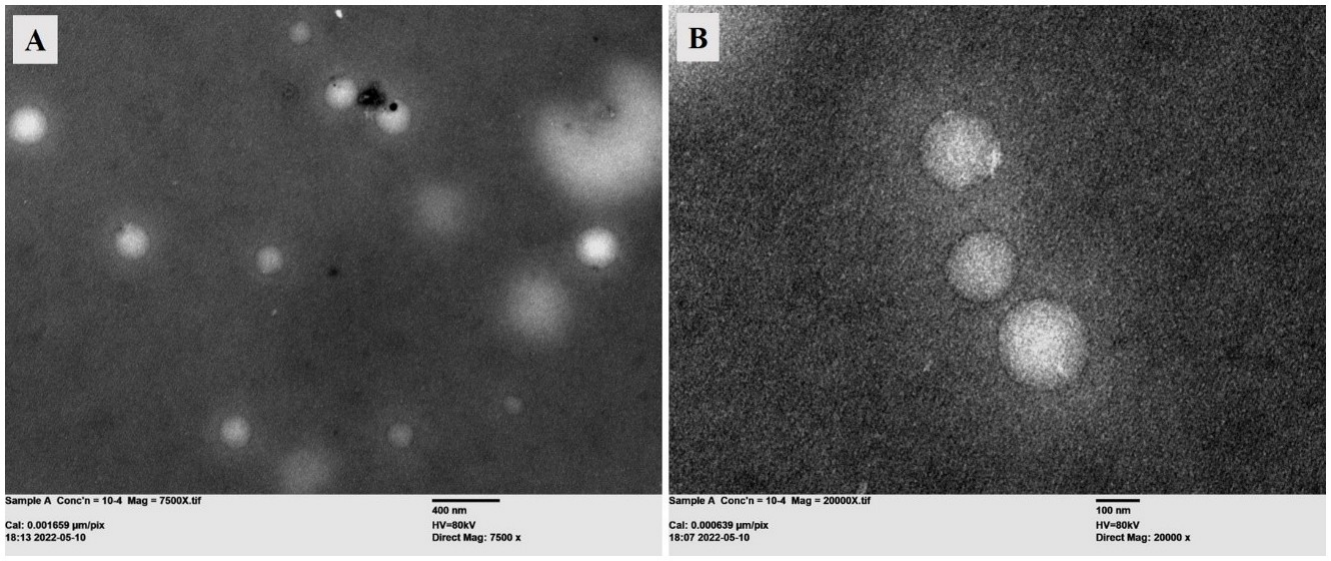
TEM analysis of 5-FU-loaded SLNs at (A) 7500X magnification (scale bar 400 nm) and (B) 20000X magnification (scale bar 100 nm).

The morphology of the optimized formulation (SLN4) was evaluated using the transmission electron microscope (Jeol, USA). The TEM analysis showed spherical-shaped nanoparticles as shown in Fig 1. The size of SLNs from TEM analysis was in accordance by the size determined by the DLS technique. The morphology of SLNs was found in accordance with already published literature [60].

#### 3.1.4. FTIR analysis

**Fig 2:**
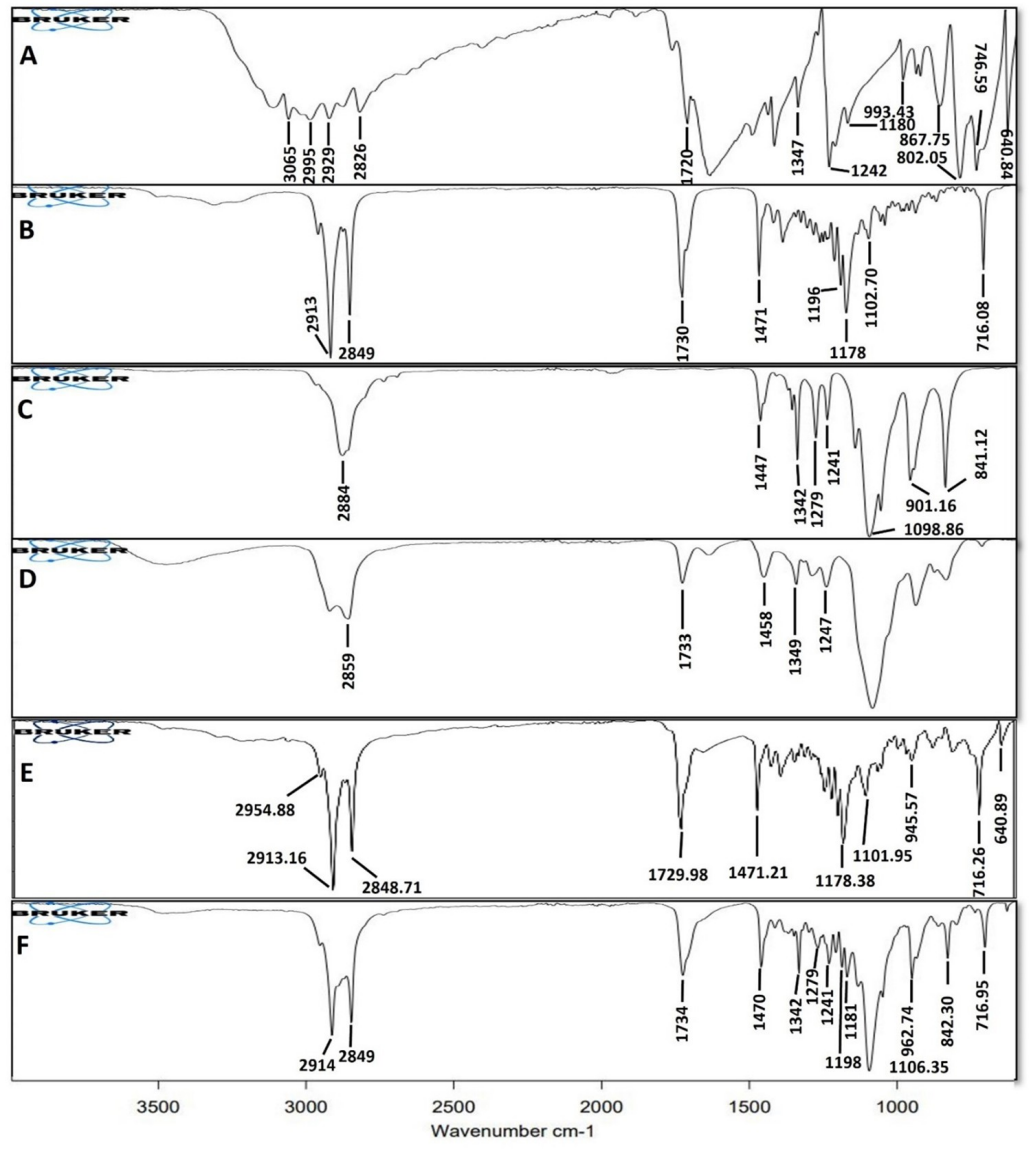
FTIR analysis of 5-FU (A), Precirol^®^ ATO 5 (B), Poloxamer 188 (C), Tween 80 (D), Physical mixture (E), and optimized formulation (F).

FTIR analysis of 5-FU, Precirol^®^ ATO 5, Poloxamer 188, Tween 80, physical mixture, and optimized formulation was performed to determine the chemical interaction between components and developed SLNs as shown in Fig 2.

5-FU shows characteristic peaks in the region between 3100 cm^-1^ and 2800 cm^-1^ due to ‒C‒H stretching, the characteristic peak at 1720 cm^-1^ was found due to ‒C = O, and peaks at 1347 cm^-1^ and 1242 cm^-1^ were found due to ‒C‒N stretching [61]. Precirol^®^ ATO 5 showed major peaks at 1730 cm^-1^ due to ‒C = O stretching and between 2913 cm^-1^ and 2849 cm^-1^ due to ‒C‒H stretching [62].

Poloxamer 188 showed characteristic peaks at 2883 cm^-1^ due to aliphatic ‒C‒H stretching and between 1447 cm^-1^ and 1342 cm^-1^ due to ‒OH bending [63, 64]. The characteristic peaks of Tween 80 at 2858 cm^-1^ were due to ‒CH_2_ stretching, and at 1733 cm^-1^ due to ‒C = O stretching [65]. Physical mixture and developed 5-FU-loaded SLNs showed characteristic peaks of individual components. The optimized formulation showed slight variation in peaks as peaks were found to be remarkably diffused. Therefore, it can be concluded that there was no chemical interaction among the formulation components and 5-FU is stable in the developed SLN system.

#### 3.1.5. pXRD analysis

**Fig 3:**
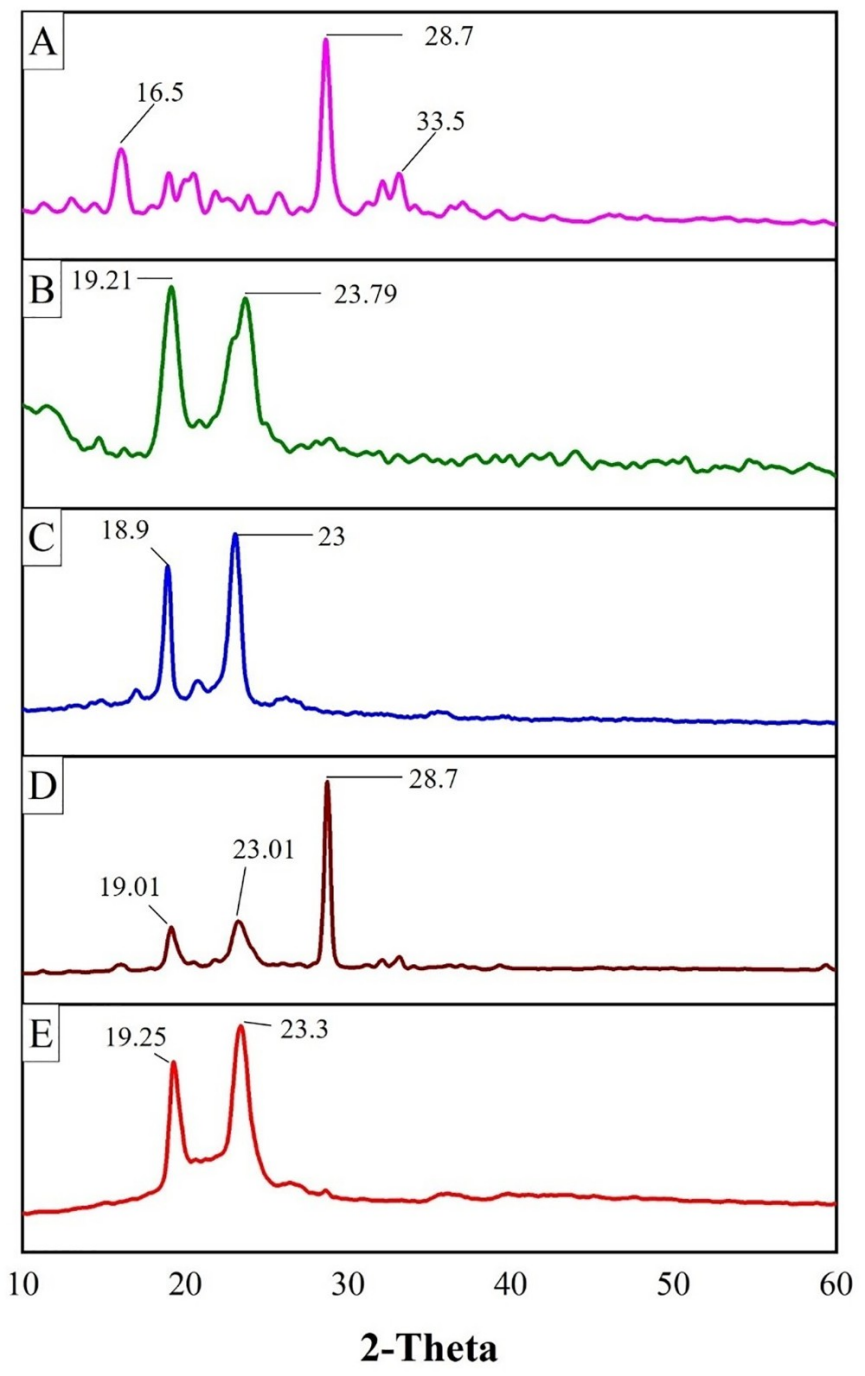
*p*XRD analysis of 5-FU (A), Precirol^®^ ATO5 (B), Poloxamer 188 (C), Physical mixture (D), and optimized formulation (E).

*p*XRD analysis of 5-FU, Precirol^®^ ATO 5, poloxamer 188, physical mixture of individual components, and the optimized formulation (SLN4) was shown in Fig 3. The *p*XRD pattern of pure 5-FU presented an intense peak at 28.7° and less intense peaks between 16.5° and 33.5° which showed the crystalline nature of 5-FU [61, 66]. The *p*XRD analysis of solid lipid Precirol^®^ ATO 5 showed characteristic peaks at 19.21° and 23.79° which represented the crystalline nature of solid lipid [67]. Poloxamer188 showed characteristic peaks at 18.9° and 23° which also showed the crystalline nature of poloxamer [63]. The *p*XRD of the physical mixture indicates the characteristics peaks of 5-FU, Precirol^®^ ATO 5 and Poloxamer 188 while The *p*XRD pattern of optimized formulation (SLN4) depicted the disappearance of the characteristic peak of 5-FU indicating the successful encapsulation of the drug in the lipid matrix and the conversion of the crystalline nature of the drug to an amorphous form. Although the characteristic peaks of solid lipid did not completely disappear or shift, rather there was seen a slight reduction in the intensity of peaks which may be due to the encapsulation of drug between the crystal lattice of lipid leading to a decrease in its crystallinity.

#### 3.1.6. DSC analysis

**Fig 4:**
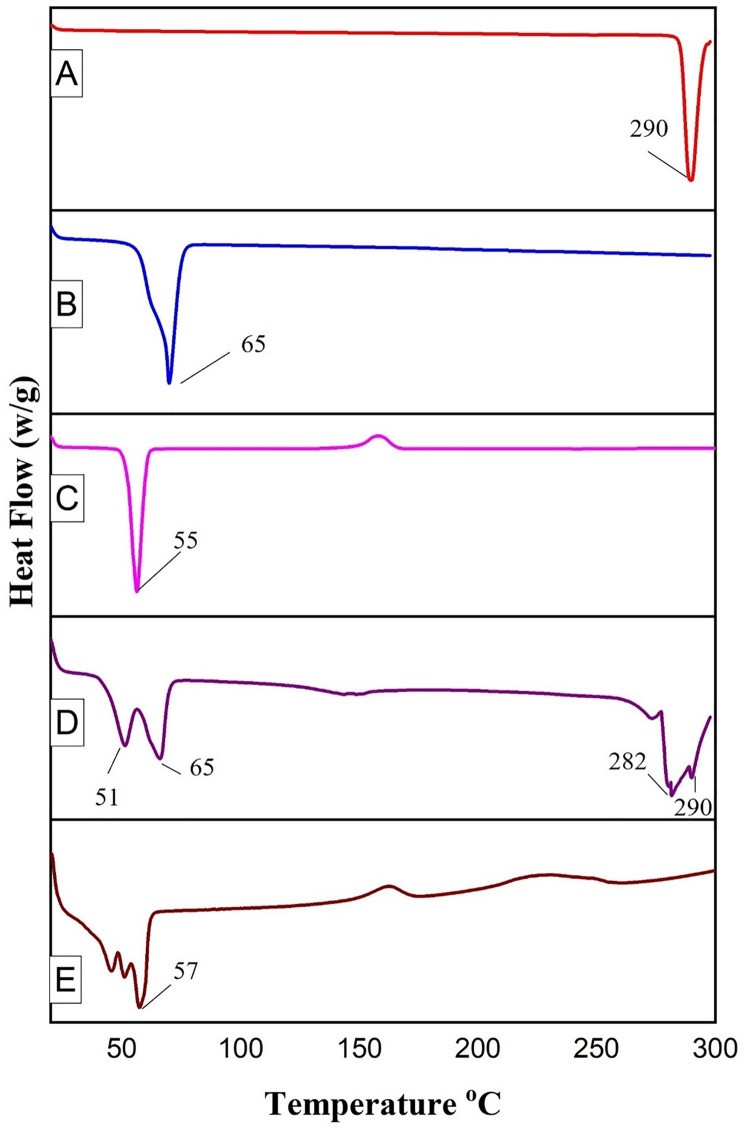
DSC analysis of 5-FU (A), Precirol^®^ ATO5 (B), Poloxamer 188 (C), Physical mixture (D), and optimized formulation (E).

DSC thermograph of 5-FU, Precirol^®^ ATO 5, Poloxamer 188, physical mixture, and an optimized formulation (SLN4) was represented in Fig 4. The DSC thermograph of 5-FU showed an endothermic peak at 290°C representing its crystalline nature [31]. The melting point of solid lipid Precirol^®^ ATO 5 was observed at 65°C [68]. The Poloxamer 188 showed an endothermic peak at 55°C [69]. The thermograph of the physical mixture indicated the characteristic peaks of 5-FU while the thermograph of lyophilized optimized formulation SLN4 did not show the endothermic peak for 5-FU around 290°C, showing that 5-FU was not in crystalline form but exists in amorphous form, the same was confirmed by *p*XRD analysis as well. The same findings have also been reported by Tran, T.H., et al., Chaves, L.L., et al., and Teixeira, M.I., et al. [70–72].

The optimized formulation shows 3 endothermic peaks around 50°C, which indicates the presence of lipid Precirol and self-assembled Poloxamer 188 on the outer surface of nanoparticles. The melting of Precirol ATO 5 in the optimized formulation was depressed showing a slight shift to lower temperature when compared to individual lipid. This depression is due to smaller particle size, higher surface area, and presence of surfactant and can be explained by the kelvin effect [73]. Kelvin determined that smaller nanoparticles would melt at a lower temperature as compared to the melting point of individual lipid as cited by Khalil *et al*., [74].

#### 3.1.7. In-vitro release studies

**Fig 5:**
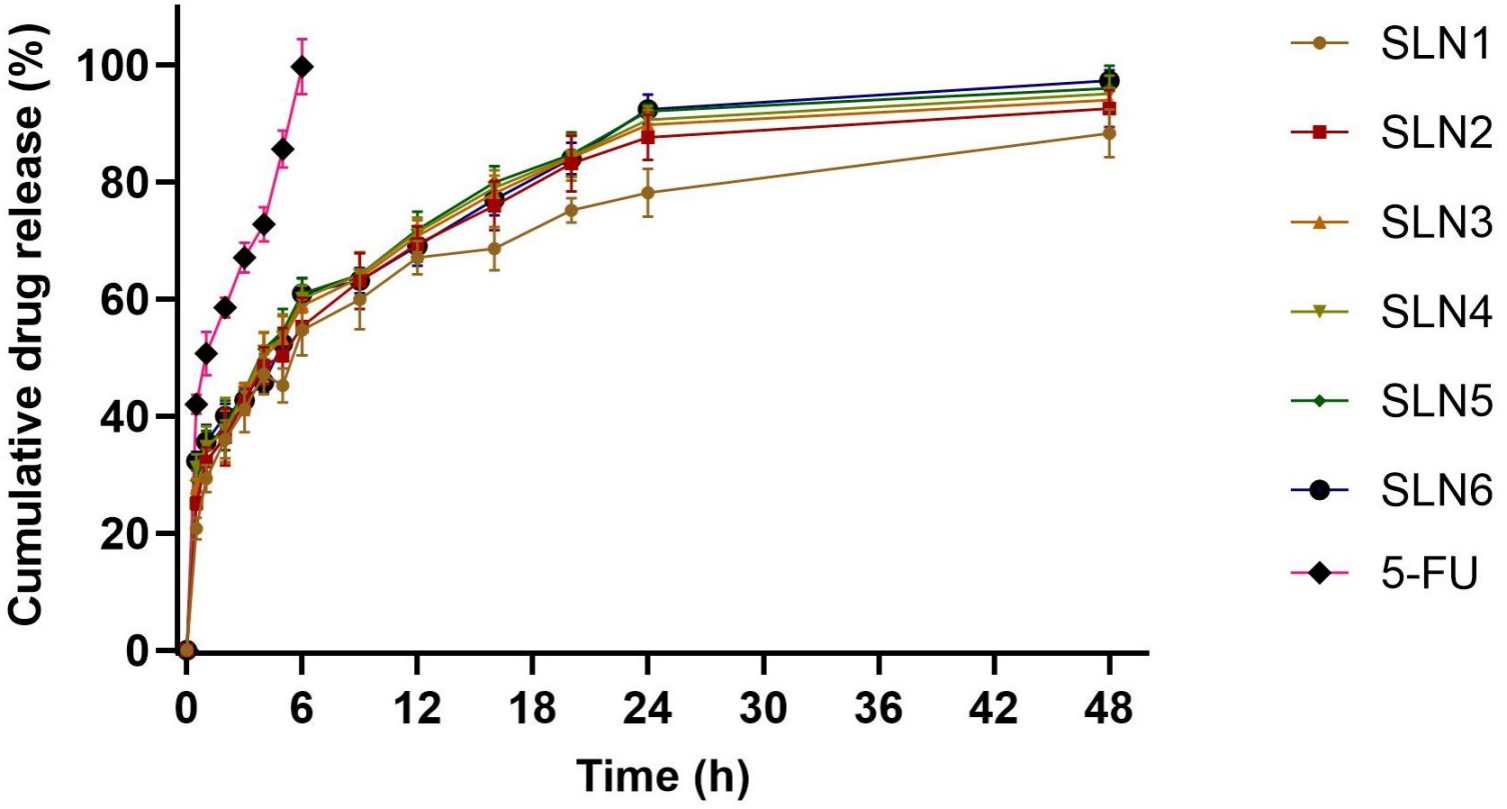
The *in-vitro* release profile of 5-FU and 5-FU-loaded SLNs in PBS pH 7.4. All results indicate mean ±SD, *n* = 3, and *p* <0.05.

The *in-vitro* release behavior of 5-FU-loaded SLNs and 5-FU drug solution were performed (Fig 5). The release of free 5-FU was rapid and almost complete from the dialysis bag within just 6 hr. The release profile of 5-FU could be used to differentiate the release pattern of 5-FU from SLNs as long as sink conditions were maintained. In comparison to the free 5-FU, the drug release profile of 5-FU from SLNs showed biphasic behavior with initial burst release of about 40–45% within the first 3 hr followed by sustained release over 48 hr. The initial rapid release may be due to the drug absorbed on the surface of SLNs [75, 76]. 5-FU being a hydrophilic drug exhibit the tendency to migrate to the aqueous phase during SLN preparation hence concentrating on or near the surface of particles exhibiting initial burst release. After initial burst release, the 5-FU from SLNs exhibited sustained release which may be due to the diffusion of the entrapped drug through the lipid matrix [77] and was found between 88–98% for all formulations over 48 hr. In the first three formulations (SLN1, SLN2, and SLN3), when the concentration of poloxamer 188 was decreased from 3% to 1.5%, the release of 5-FU was increased from 88% to 94% due to decreased particle size and increased surface area of nanoparticles [78]. In the next three formulations (SLN4, SLN5, and SLN6) when the concentration of poloxamer 188 was optimized, the release was found between 95 to 98% over 48 hr.

#### 3.1.8. Kinetic modeling

The kinetic analysis modeling data of drug release from different formulations of 5-FU-loaded SLNs were shown in Table 2. Comparing the results of all models it was found that the release kinetics were best fitted into Korsmeyer-Peppas Model. The value of “*n*” for all the 5-FU-loaded SLNs was in the range of 0.276–0.292, less than 0.5, and depicted Fickian diffusion release behavior [79]. The fitted curves/graph are shown in supplementary data.

**Table 2:** Kinetic modeling of 5-FU-loaded SLNs.

Code	Zero Order	First Order	Higuchi Model	Korsmeyer-Peppas	
	R^2^	R^2^	R^2^	R^2^	*n*
SLN1	0.5867	0.7423	0.7501	0.9860	0.289
SLN2	0.5636	0.8240	0.7575	0.9823	0.292
SLN3	0.6223	0.8293	0.7384	0.9805	0.286
SLN4	0.7279	0.8009	0.7053	0.9811	0.276
SLN5	0.6849	0.8228	0.7192	0.9818	0.280
SLN6	0.6357	0.7811	0.7310	0.9789	0.283

### 3.2. In-vitro cytotoxicity

**Fig 6:**
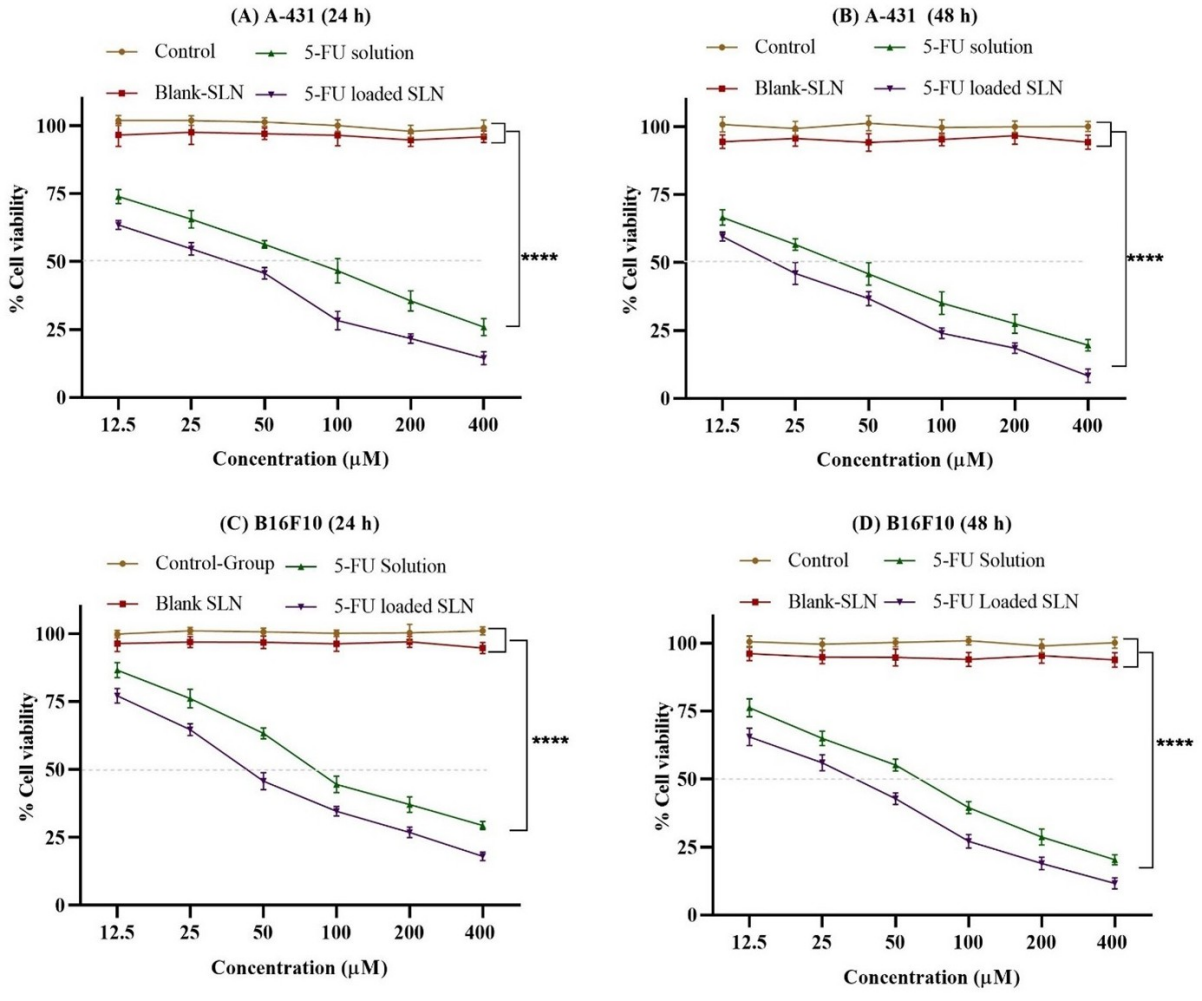
Cytotoxicity profile of blank SLN, 5-FU drug solution and 5-FU-loaded SLN on A-431 after 24 h (A), 48 h (B) and B16F10 after 24 h (C) and 48 h (D). All results indicate mean ±SD, *n* = 3, p****<0.0001 (Two-way ANOVA with Tukey’s Post hoc test).

The *in-vitro* cytotoxicity of the free drug (5-FU), blank SLNs, and 5-FU-loaded SLNs on B16F10 and A-431 cells was evaluated after 24 hr and 48 hr (Fig 6). The results showed that blank SLNs have negligible cytotoxic effects indicating the biocompatibility of the formulation. After 24 hr and 48 hr of incubation, the 5-FU-loaded SLNs showed greater cytotoxicity to both B16F10, and A-431 cells as compared to 5-FU. As seen in Fig 6, the 5-FU-loaded SLNs has a higher cytotoxic effect on B16F10 and A-431 cells than pure 5-FU at all concentration and time intervals and these results are in accordance with the previous studies [80, 81].

### 3.3. Quantitative uptake by flow cytometry

**Fig 7:**
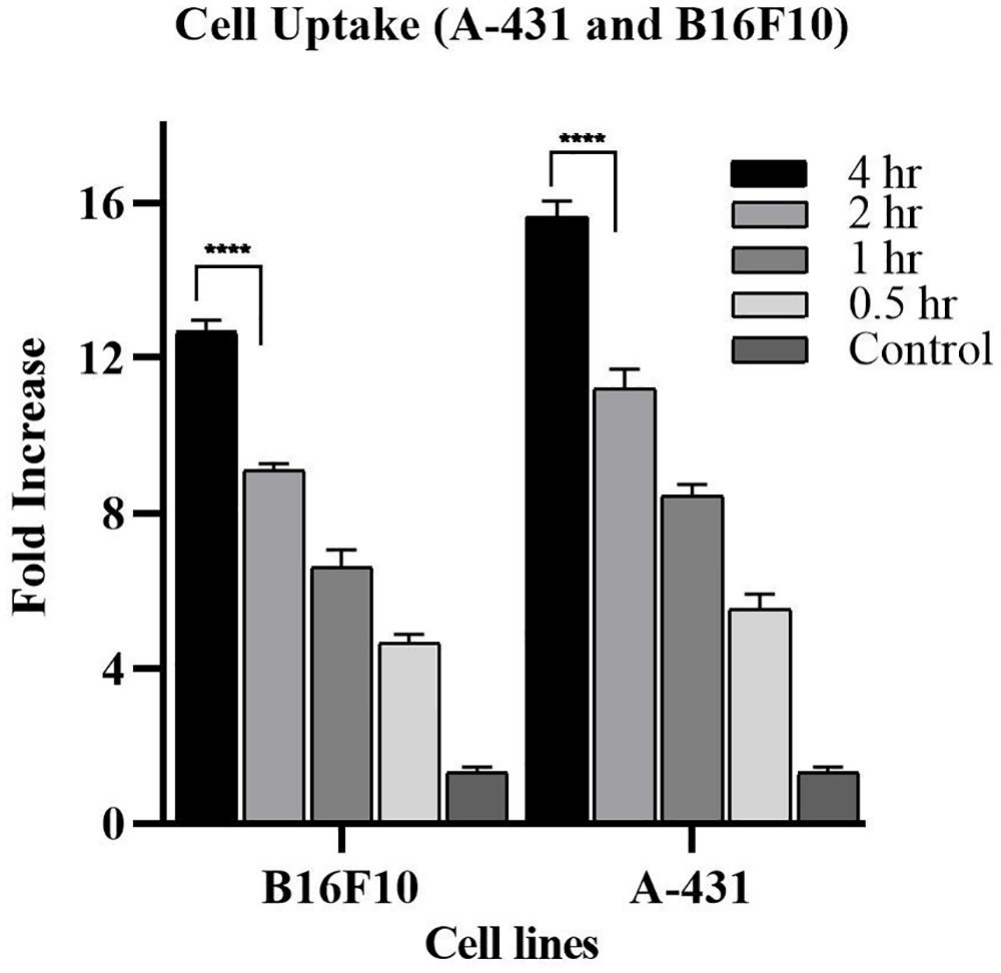
Cell uptake of SLNs as compared to control after 0.5, 1, 2 and 4 hr. All results indicate mean ± SD, *n* = 3. ****p < 0.0001 (After Two-way ANOVA with Tukey’s post hoc test).

The quantitative cellular uptake of SLNs was determined by flow cytometry. The results of flow cytometry analysis showed that SLNs showed 12.66-fold and 15.63-fold increase in uptake in B16F10, and A-431 cells respectively as compared to control after 4 hr (Fig 7).

### 3.4. Qualitative uptake by fluorescence microscopy

**Fig 8:**
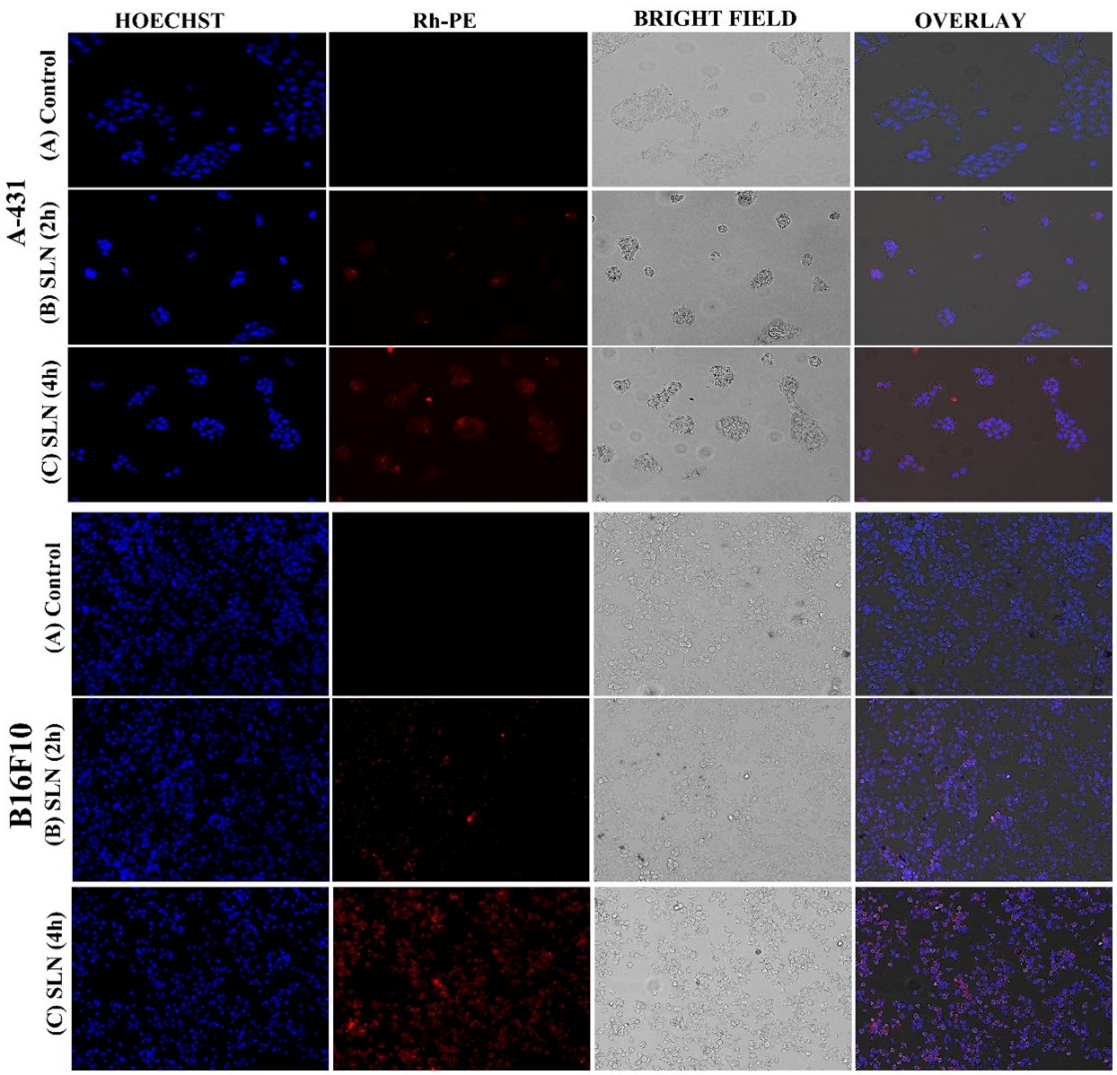
Qualitative cellular uptake studies of control vs treated cells with SLNs.

Fluorescence microscopy was used to observe the qualitative cellular uptake/cellular association of nanoparticles at 2 hr and 4 hr time intervals. Under fluorescence microscopy, Hoechst produces blue color after interaction with the DNA of the cells and Rh-PE produces a red color. Fig 8 represents more uptake/association observed by both A-431 and B16F10 cells when treated with SLNs as compared to control. It was also observed that uptake is time-dependent and might be that SLNs were taken up by the cells via endocytosis resulting in high cellular uptake [82, 83].

### 3.5. Acute toxicity study

**Fig 9:**
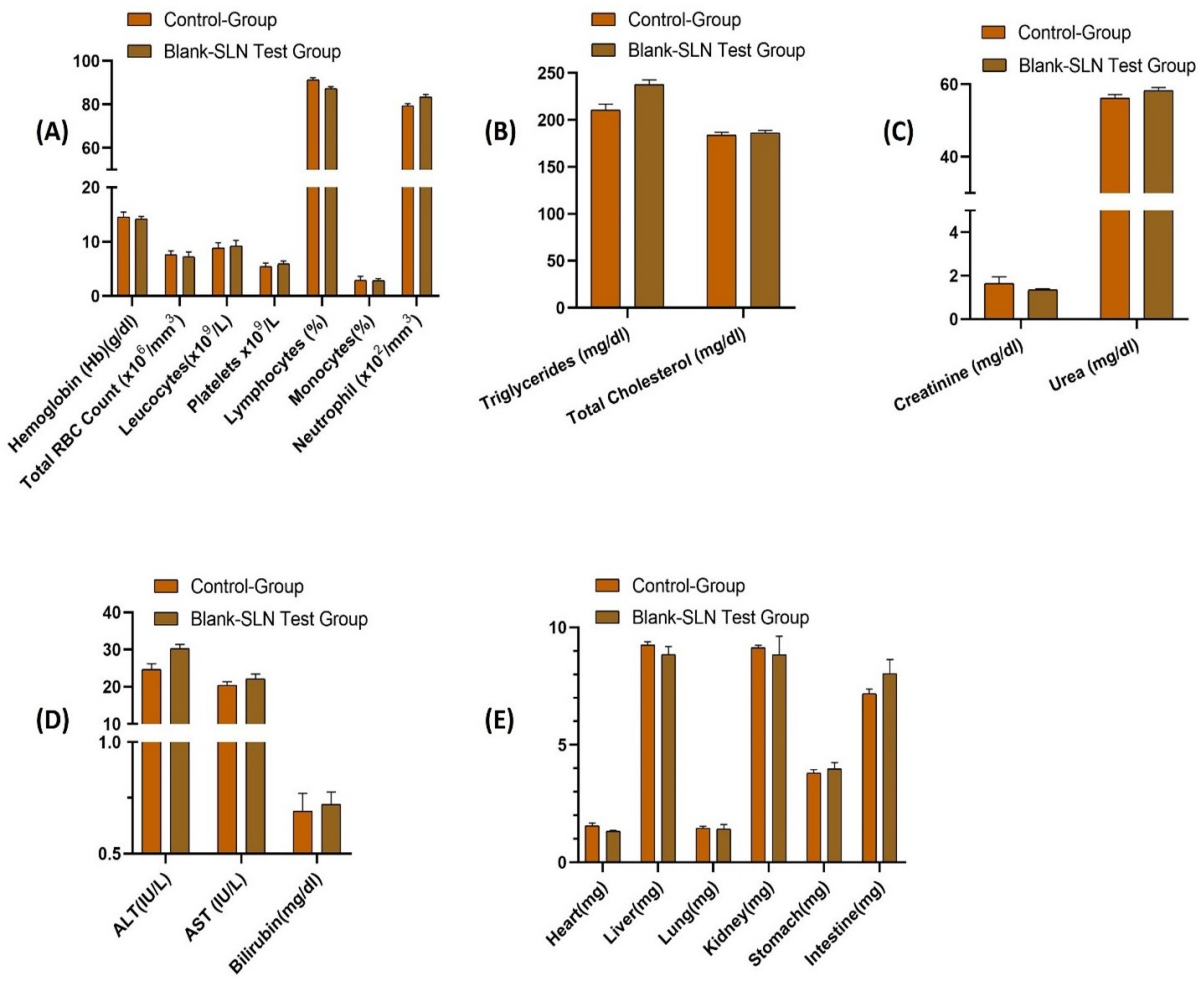
Analysis of biochemical and hematological parameters in the control group and blank SLN4 (test group). (A) hematological parameters, (B) lipid profile, (C) renal function test, (D) liver function test, (E) organ weight. All results indicate mean ± SD, (*n* = 6).

**Fig 10:**
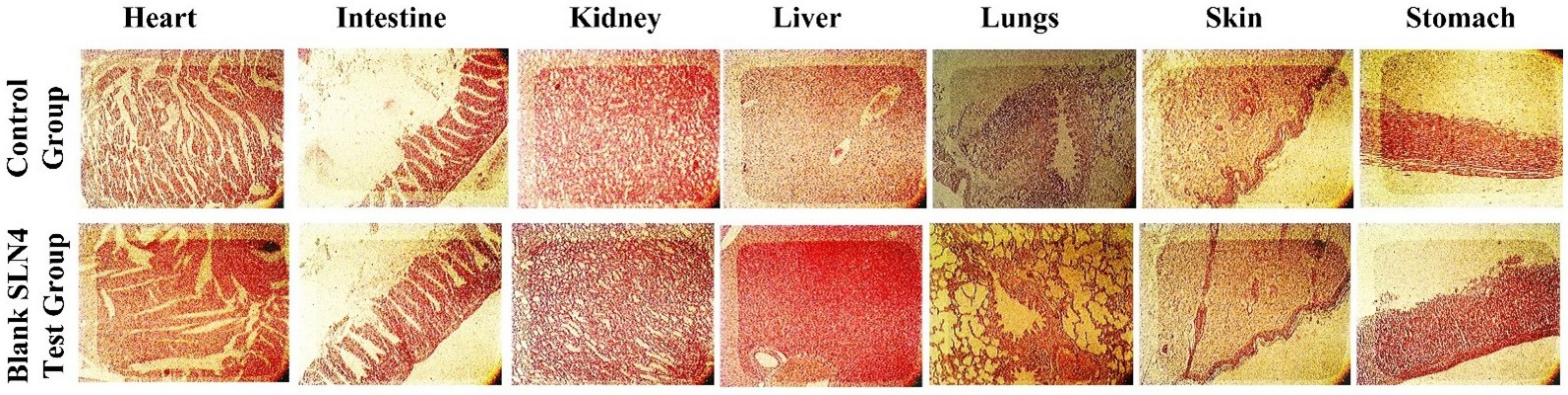
Histopathological analysis of Heart, intestine, kidney, liver, lungs, skin and stomach of control group animals and blank SLN4 test group animals.

The safety and biocompatibility of the developed SLNs (blank SLN4) were determined by an acute toxicity study. During the study, no signs of illness after the physical examination was found and the water and food intake remained normal for 14 days in both control and blank SLN4 test groups. Different pathological markers including liver function, kidney function, hematology, and lipid profile were checked to determine the safety of the developed SLNs and were compared with a control group. The developed SLNs (blank SLN4s) have negligible effects when compared with the control (Fig 9A–9D) (results also shown in the supplementary data). There were no observable variations found in both the organ weight of the control and SLNs test group (Fig 9E). Moreover, the histopathological evaluation revealed that there were no signs of toxicity, lesions, distortion, and disruption of tissues in the vital organs (Fig 10). In brief, the acute toxicity study depicted that the developed SLNs were safe and did not show any toxicity.

### 3.6. Characterization of 5-FU-loaded SLNs gel

#### 3.6.1. Physical appearance pH, spreadability, and rheological analysis

The developed gels (5-FU-loaded SLNs gel and 5-FU plain gel) were smooth in texture, uniform, and clear and the pH of both gels was maintained at 5.5 indicating a slightly acidic nature. The pH between (4–6) is considered suitable for topical formulation because it favors the intact delivery of nanoparticles across the skin and avoids skin irritation and incompatibility [84, 85]. The spreadability of gel is determined to evaluate its uniform application of gel and was found 4.49 ± 0.23 cm for 5-FU-loaded SLNs gel and 5.2 ± 0.18 cm for 5-FU plain gel and considered suitable for topical application[86, 87].

**Fig 11:**
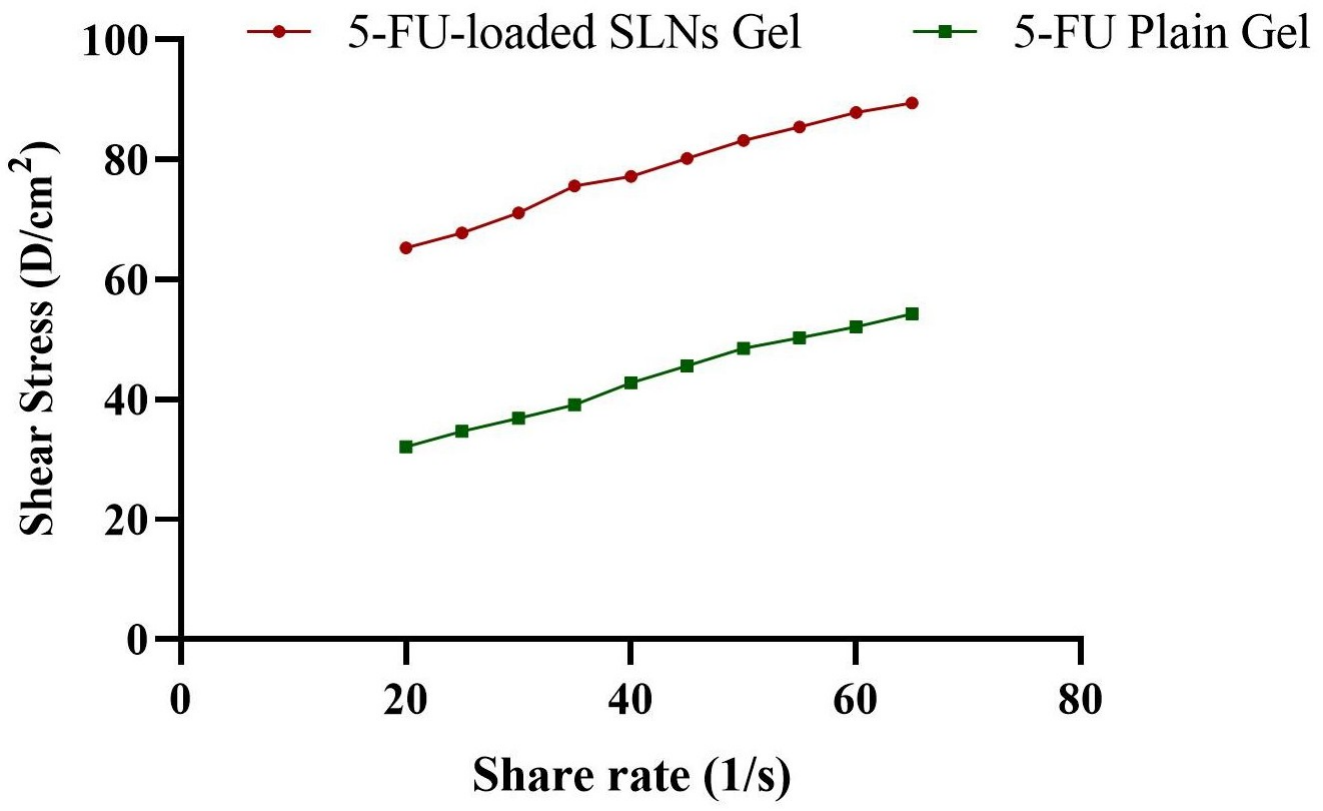
Rheograms of 5-FU plain gel and 5-FU-loaded SLNs gel.

Fig 11 indicates the rheogram indicating shear stress and a shear rate of freshly prepared 5-FU plain gel and 5-FU-loaded SLNs gel. Rheological parameters are important to determine because the spreading behavior of gel as well as diffusion of the drug from gel is based on these parameters [88]. Both the gels showed shear thinning behavior as a function of shear rate, and this behaviour is ideal for topical application [89].

#### 3.6.2. Ex-vivo permeation study

**Fig 12:**
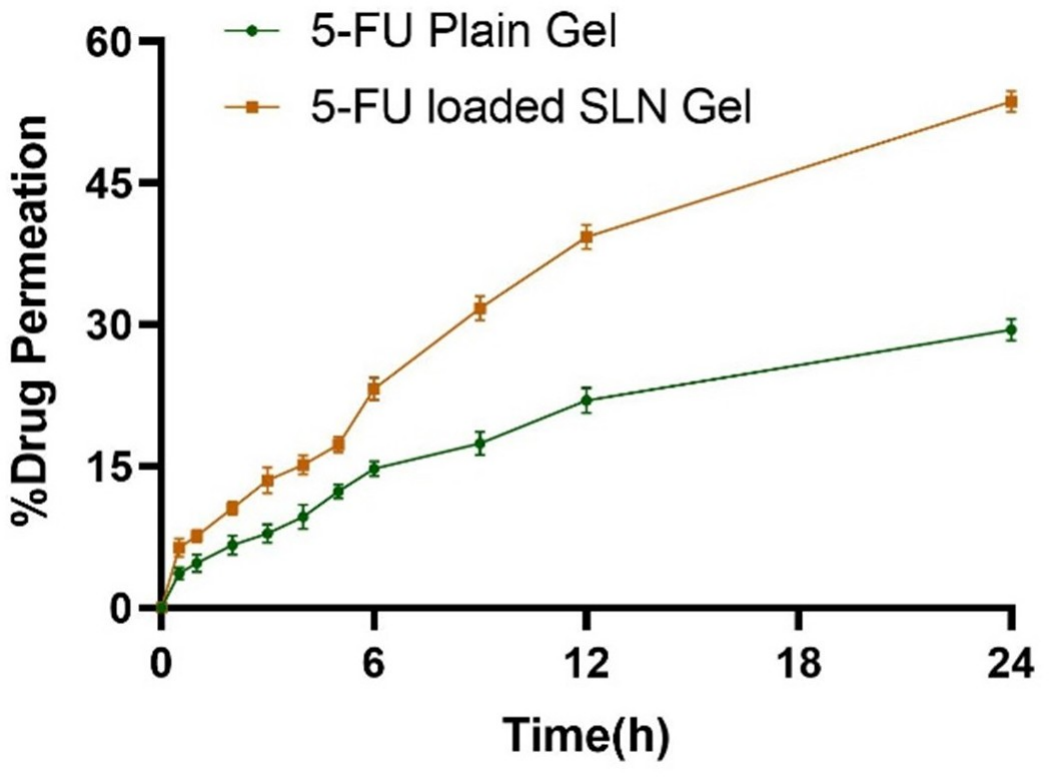
*Ex-vivo* permeation analysis of 5-FU-loaded SLNs gel and 5-FU plain gel.

Fig 12 represents the % drug permeation from 5-FU-loaded SLNs gel and 5-FU plain gel and the results of permeation data were represented in Table 3. The permeation of 5-FU was found to be enhanced from 5-FU-loaded SLNs (53.66% ± 1.103) in comparison with 5-FU plain gel (29.46% ± 1.15) across Wistar rat skin over 24 h at pH 5.5. The enhanced skin permeation of 5-FU-loaded SLNs gel is mainly due to the larger surface area and smaller size of SLNs that interface with skin corneocytes, providing a more occlusive effect and increasing hydration of stratum corneum as compared to plain gel [90]. The permeability flux of 5-FU-loaded SLNs gel was found to be 16.86 μg/cm^2^/h and the co-efficient of permeability was found to be 0.00741 cm/h while the permeability flux of 5-FU plain gel was found to be 7.8 μg/cm^2^/h and the co-efficient of permeability was observed at 0.0036 cm/h. Likewise, the enhancement ratio of 5-FU-loaded SLNs gel was found 2.13±0.076 representing a manifold increase in skin permeation through 5-FU-loaded SLNs. The statistical analysis of permeation studies of 5-FU-loaded SLNs gel with the 5-FU plain gel showed a significant difference (*p<*0.05) when the t-test was applied.

**Table 3:** Permeation analysis of 5-FU-loaded SLNs gel and 5-FU plain gel.

Formulation	Flux (μg/cm^2^.hr)	Papp (cm/h)	%Drug Permeated	%ER	%TE
5-FU-loaded SLNs gel	16.86	0.0074	53.66%±1.103	2.13±0.076	2.54±0.03
5-FU Plain gel	7.8	0.0036	29.46%±1.15		

#### 3.6.3. Skin retention study

The amount of drug retained in the skin was estimated by extracting the drug from skin tissues. The 5-FU-loaded SLNs gel exhibited more retention of 5-FU (36.75% ± 0.298) as compared to 5-FU plain gel (14.47% ± 0.43). Moreover, the target efficiency of 5-FU-loaded SLNs was found to be 2.54 indicating more drug retention as compared to 5-FU plain gel (Table 3). This was due to the formation of a dense occlusive layer of solid lipids that melts and penetrates through the skin due to the suitable physicochemical properties of SLNs [91].

## 4. Conclusion

In the present study, 5-FU-loaded SLNs were successfully prepared and characterized for physicochemical properties to optimize size distribution for enhanced permeability and retention effects at the cancer site. The developed 5-FU-loaded SLNs provided sustained release to maximize the therapeutic effect and minimize dose related toxicity of 5-FU. The developed 5-FU-loaded SLNs depicted enhanced cytotoxicity to skin melanoma and squamous cell carcinoma cells in a concentration and time-dependent manner. The acute toxicity study unveiled that developed SLNs are safe carriers for the delivery of the chemotherapeutic agent. The developed 5-FU-loaded SLNs gel showed increased skin permeation and retention compared to plain gel and could be used as a topical drug delivery system for the anticancer effect against skin melanoma and squamous cell carcinoma after authentication through *in-vivo* studies.

## Institutional review board statement

The acute toxicity study was performed according to the Organization for Economic Co-operation and Development (OECD) guidelines. The approval of the study was taken from the Pharmacy Animal Ethics Committee (PAEC), Institutional Ethical committee under Reference No: 33-2021/PAEC.
